# Non-invasive biomarkers for monitoring the immunotherapeutic response to cancer

**DOI:** 10.1186/s12967-020-02656-7

**Published:** 2020-12-09

**Authors:** Sabah Nisar, Ajaz A. Bhat, Sheema Hashem, Santosh K. Yadav, Arshi Rizwan, Mayank Singh, Puneet Bagga, Muzafar A. Macha, Michael P. Frenneaux, Ravinder Reddy, Mohammad Haris

**Affiliations:** 1 Functional and Molecular Imaging Laboratory, Cancer Research Department, Sidra Medicine, P.O. Box 26999, Doha, Qatar; 2 Department of Nephrology, AIIMS, New Delhi, India; 3 Department of Medical Oncology, Dr. B. R. Ambedkar Institute Rotary Cancer Hospital (BRAIRCH), AIIMS, New Delhi, India; 4 Department of Diagnostic Imaging, St Jude Children’s Research Hospital, 262 Danny Thomas Place, Memphis, TN USA; 5 Watson-Crick Centre for Molecular Medicine, Islamic University of Science and Technology, Awantipora, Jammu & Kashmir India; 6 Academic Health System, Hamad Medical Corporation, Doha, Qatar; 7 Center for Magnetic Resonance and Optical Imaging, Department of Radiology, Perelman School of Medicine, University of Pennsylvania, Philadelphia, USA; 8 Laboratory Animal Research Center, Qatar University, Doha, Qatar

**Keywords:** Cancer metabolism, Immunotherapy, T cells, Tumor microenvironment, Imaging biomarkers

## Abstract

Immunotherapy is an efficient way to cure cancer by modulating the patient’s immune response. However, the immunotherapy response is heterogeneous and varies between individual patients and cancer subtypes, reinforcing the need for early benefit predictors. Evaluating the infiltration of immune cells in the tumor and changes in cell-intrinsic tumor characteristics provide potential response markers to treatment. However, this approach requires invasive sampling and may not be suitable for real-time monitoring of treatment response. The recent emergence of quantitative imaging biomarkers provides promising opportunities. In vivo imaging technologies that interrogate T cell responses, metabolic activities, and immune microenvironment could offer a powerful tool to monitor the cancer response to immunotherapy**.** Advances in imaging techniques to identify tumors' immunological characteristics can help stratify patients who are more likely to respond to immunotherapy. This review discusses the metabolic events that occur during T cell activation and differentiation, anti-cancer immunotherapy-induced T cell responses, focusing on non-invasive imaging techniques to monitor T cell metabolism in the search for novel biomarkers of response to cancer immunotherapy.

## Introduction

Cancer immunotherapy has emerged as a treatment method for various cancers by targeting the mechanisms that govern the interplay between tumor microenvironment and immune cells. The general premise of immunotherapy for cancer is to stimulate, enhance, or improve the antitumor immune response of the host. Despite the advances in immunotherapy, only some patients showed a significant clinical benefit while the majority of patients depicted substantial side effects. Therefore, the immunotherapies must be targeted to the patients who are likely to benefit, suggesting an urgent need to identify biomarkers that can direct patient selection and help determine the response to treatment at an early stage. Non-invasive molecular imaging has become an essential diagnostic modality in cancer management. Because of molecular imaging’s potential to test biological processes with high precision in vivo non-invasively at the whole-body level, it is of great importance to improve these technologies to direct treatment under many oncological conditions. Several immunotherapeutic techniques are employed in cancer therapy, including modulation of T cell activity through adoptive cell transfer (ACT), monoclonal antibodies (mAbs), checkpoint inhibitors, and cancer vaccines [1–3]. The common denominator for successfully implemented immunotherapies in the clinic is their ability to stimulate or increase cytotoxic T cells' infiltration into the tumor. Thus, in vivo imaging technologies that target T cell responses in patients are powerful tools for further development of immunotherapy. The following sections provide an overview of T cells' metabolism and the factors affecting these metabolic pathways and how non-invasive imaging techniques can evaluate immunotherapeutic efficacy by targeting these factors.

## T cell metabolism

**Fig. 1:**
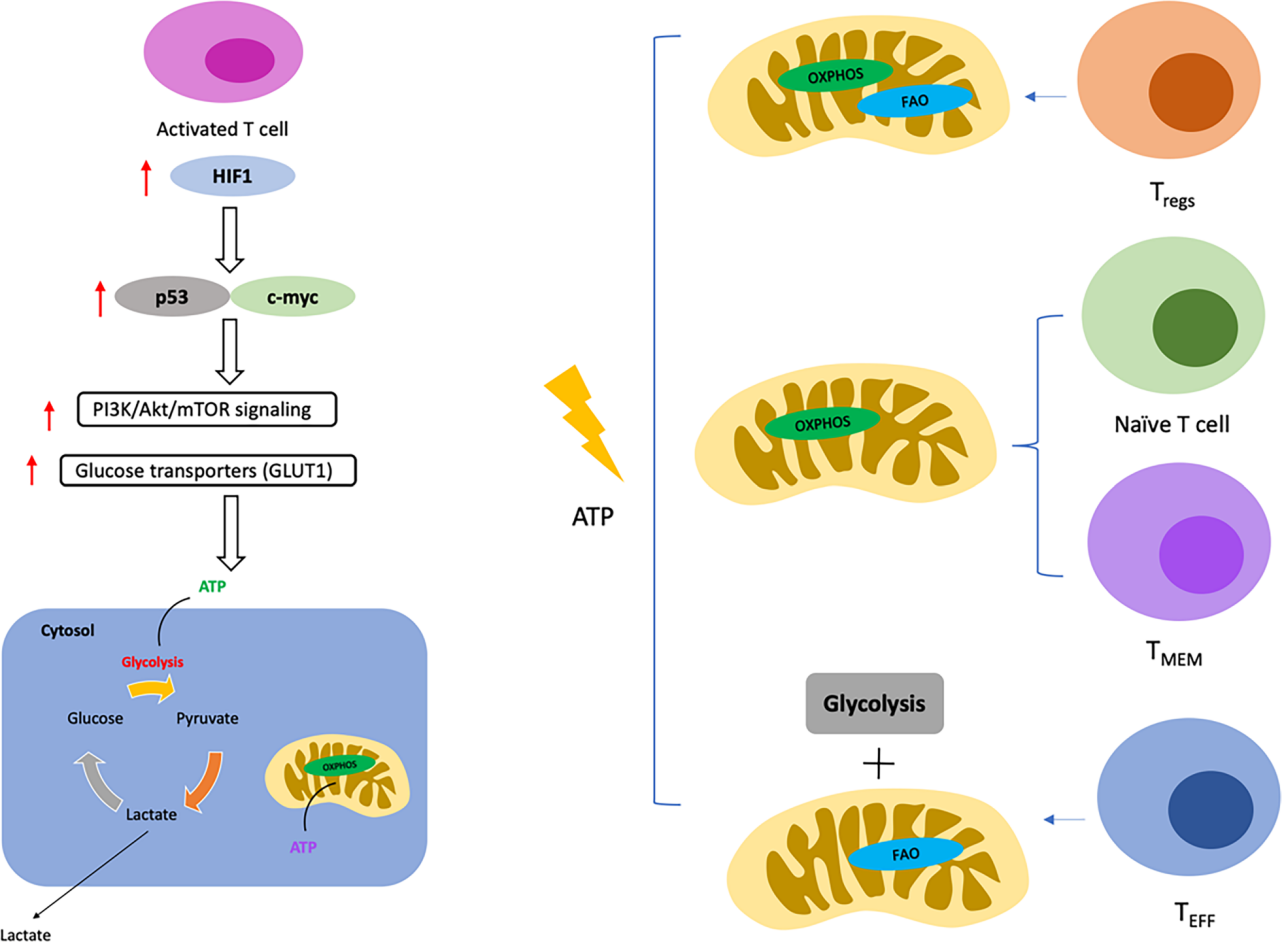
Different mechanisms of energy production in T cell subtypes. Naïve T cells and memory T cells (T_MEM_) mainly generate ATP via oxidative phosphorylation (OXPHOS). Regulatory T cells (T_regs_) produce ATP through OXPHOS and fatty acid oxidation (FAO), while, the effector T cells (T_EFF_) mainly rely on glycolysis and FAO for energy production

The tumor microenvironment (TME) plays an important role in the successful outcome of T cell-mediated adaptive immunotherapy against tumor. TME is a very dynamic and complex ecosystem comprising cellular components (fibroblasts, immune cells, endothelial cells, pericytes, and adipocytes), extracellular matrix proteins (collagen, elastin fibres, fibronectins, proteoglycans, hyaluronic acid, osteopontin, periostin and SPARC) and secretory factors including cytokines, chemokines and many growth factors. The TME also contains immunosuppressive cells such as natural killer (NK) cells, regulatory T (Treg) cells, dendritic cells (DCs), myeloid suppressor cells (MDSC), and tumor-associated macrophages (TAMs), whose role is to prevent effective antitumor immune responses [4–6]. The TME plays an important role in inducing stressful conditions and the tumor's immunosuppressive environment, resulting in metabolic reprogramming and increased plasticity in cancer cells, known to be a key mechanism of drug resistance. During metabolic reprogramming, the malignant cells undergo significant changes in metabolism compared to normal cells to meet the energy demands for rapid proliferation and better survival in the unfavorable conditions of the TME. The major change in cancer cells to compensate for the lower efficiency of glycolysis energy generation is the upregulation of glucose transporters, in particular Glut1, Glut2, Glut3, and Glut4, to uptake more glucose [7–9]. Another change is the upregulation of several glycolytic enzymes due to the high transcriptional activity of c-Myc and HIF-1α and insufficient p53-mediated control [10–14]. The TME's immune cells often compete with cancer cells for the nutrients available, and the type of nutrients present in the TME alter the function and differentiation of immune cells. The differentiation program of macrophages, DCs, and T cells are guided by fluctuations in amino acids’ accessibility, fatty acids, and glucose [15–21]. Accumulation of lactate and other metabolic products in the TME due to increased cancer cell metabolism suppresses the immune cell activity, including altered differentiation of dendritic cell and macrophage and impaired anti-tumor activity of T cells [22]. Metabolic reprogramming in T cells is also controlled largely by key signaling receptor events, growth factors, and cytokines. All of these events can alter T cell’s functional fate by post-translation modifications (PTMs) or epigenetic remodeling [23–29]. Throughout their development, T cells undergo a series of quiescence and activation events, which helps to maintain them in the proper functional state when required. The main signals that push T cells out of quiescence are pathogenic antigens, tumor cell antigens or inflammatory signals, leading to rapid regulation of gene expression, increased metabolism, increased transcriptional activity, and rearrangement in the cytoskeleton, which are important for the growth and differentiation of T cell subsets. These various changes in T cells result in the attainment of diverse immune functions such as augmented production of cytokines, cytotoxic molecules and amplified T helper cell activity. Regulation of cellular metabolism is intimately integrated into this activation program. Activation of effector T cells (T_EFF_) caused by the ligation of T Cell Receptor (TCR) to Major Histocompatibility Complex (MHC) bound antigens accompanied by secondary signals from the involvement of costimulatory proteins induces naive T cells to undergo remodeling of their metabolic system to facilitate anabolic growth and bio-mass accrual. The uncoupling of glycolysis manifests these changes in T cell metabolism from glucose activation due to reduced activity of the Pyruvate Dehydrogenase (PDH) enzyme complex responsible for the conversion of Pyruvate to Acetyl CoA. This inhibition is not due to reduced oxygen availability and thus resulting in the generation of lactate from pyruvate by the enzyme lactate dehydrogenase [30, 31]. Although OXPHOS is dramatically more efficient than aerobic glycolysis in ATP generated per glucose molecule used, the metabolic intermediates provided by aerobic glycolysis are critical to cell growth and proliferation and to maintain the redox equilibrium (NAD + /NADH) (Fig. 1) [12, 32, 33]. The high energy demands are met by an increase in glucose uptake and glycolysis rate, associated with the upregulation of the pentose phosphate pathway (PPP) and glutaminolysis [34–38]. Such metabolic events during T cell activation lead to the expression of transcription factors like HIF-1α and c-Myc, which regulate the metabolic activity of T cells and subsequently control the T_EFF_ cell functions [13, 14]. The metabolism of fatty acids also plays an important role in the differentiation of various subsets of T cells. De novo fatty acid synthesis (FAS) and fatty acid uptake are key characteristics of T_EFF_ cells, while the mobilization and use of stored esterified fatty acids synthesized from glucose is a feature of T memory cells (T_MEM_) [39]. T_MEM_ cells are a dormant cell population mainly utilizing OXPHOS for energy production, but after antigen rechallenge during reinfection, there is a rapid surge in OXPHOS as well as glycolysis promoting a recall response. Notably, the competition between de novo FAS and FA uptake also regulates the differentiation state among Treg and Th17 cells [25, 40]. The important cytokines that are involved during metabolic reprogramming of T cells are IL-7 and IL-2. IL-7 increases the expression of the antiapoptotic protein Bcl-2, thereby safeguarding the naive T cells that are in the quiescent state from undergoing apoptosis, and knockdown of IL-7 or the IL-7Rα chain in mice leads to developmental defects in T cells [41–44]. IL-7 utilizes JAK3–STAT5 signaling but can also stimulate the PI3K pathway to induce its effects [45, 46]. IL-7 also participates in glucose uptake and the transcriptional regulation of the Hexokinase gene (HKII) [47]. Binding of IL-2 to the IL-2 cytokine receptor followed by costimulatory protein ligation induces glycolysis transition by enhancing the expression of nutrient transporters involved in mobilizing nutrients and activating mTOR, a crucial metabolic signaling regulator [48–50]. Thus, there are highly intricate changes driven by metabolism in the diverse T cell subsets during differentiation, and targeted therapeutic intervention of such metabolic signaling pathways could enhance the impact of immunotherapy.

### Factors affecting T cell metabolism

**Fig. 2:**
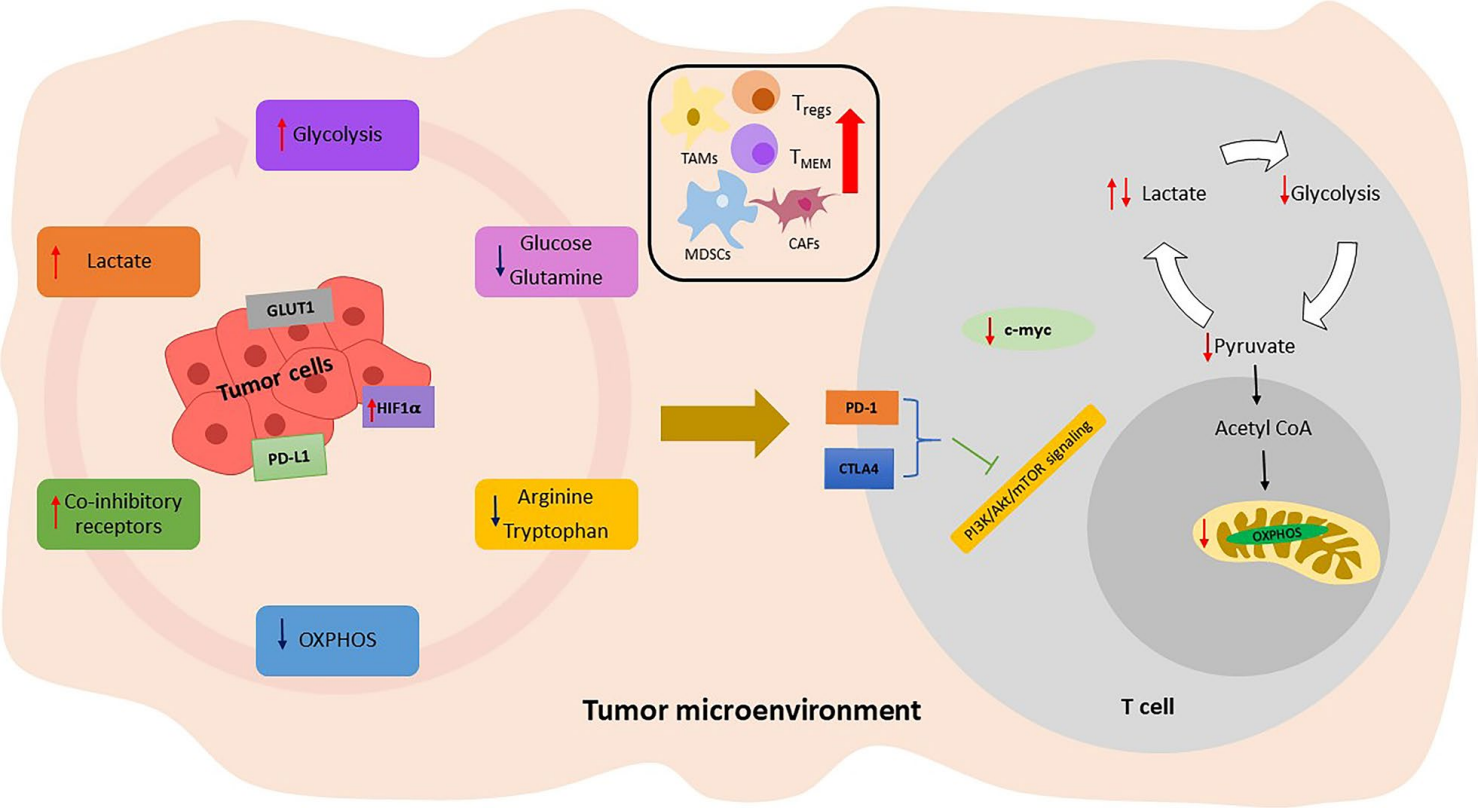
Factors affecting T cell metabolism in tumor microenvironment (TME). Nutrient competition (glucose and glutamine), amino acid depletion (arginine and tryptophan), increased acidity due to high lactate production impairs T_EFF's_ functioning_._ Also, these factors in the TME causes an increase in inhibitory cells such as T_regs_, T_MEM_, cancer-associated fibroblasts (CAFs), myeloid-derived suppressor cells (MDSCs), and tumor-associated macrophages (TAMs) to maintain an immunosuppressive TME

The reduced blood vasculature in the TME, together with the increased metabolic activity of cancer cells, can cause nutritional deprivation (Fig. 2) [51]. These TME conditions impair signaling mediated by TCR and other metabolic pathways such as glycolysis, fatty acid synthesis, uptake, and amino acids’ metabolism, resulting in abridged tumor-specific T cell antitumor effector functions. On the other hand, Treg cells which rely primarily on FAO [31] adapt to the hostile conditions of TME and thus induce immune suppressive properties on tumor-specific T cells. The accumulation of metabolites such as glycolytic lactate and amino acid metabolism kynurenine within hyperactive cancer cells can inhibit the activation and cytolytic function of T cells [18]. In the case of hypoxia, tumor cells can significantly affect the T cell metabolism by increased development of lactate within the TME. High lactate in TME decreases both T cell proliferation and cytokines production by reducing glycolysis [52]. Hypoxia-induced HIF-1α aids the development and maintenance of Treg cells [53] and regulates the expression of PD-L1 in MDSCs, causing suppression of T_EFF_ cell function in the TME [54]. Under hypoxic conditions, HIF-1α causes increased transcription of GLUTs and lactate dehydrogenase A (LDHA), thus growing accumulation of lactate [55].

Given that glucose is essential for T_EFF_ cells’ survival and proliferation, nutrient deprivation causes inhibition of mTOR activity, which is critical for T cell metabolism [56, 57]. Glucose and essential amino acids such as arginine, tryptophan, and glutamine required by the immune cells are also depleted in the TME, leading to the anergic status of cytotoxic T cells [58]. T cell activation also depends on extracellular glutamine [59]. Glutamine undergoes anaplerosis to produce α-ketoglutarate which enters TCA cycle for the generation of citrate and pyruvate. This process ensures that the metabolites removed from the TCA cycle are replaced to maintain the integrity of the TCA cycle function by T_EFF_ cells [60]. Also, constant antigen exposure leads to chronic T cell activation and causes T cell exhaustion, thereby reducing its effector functions [61]. Chronically activated T cells express immune-inhibitory receptors like PD-1 and CTLA4. The expression of the PD-1 receptor on tumor-infiltrating lymphocytes (TILs) is associated with an exhausted TIL phenotype with impaired effector function, thus suggesting an essential role of PD-1 in suppressing T_EFF_ cell function [62]. The increased CTLA4 levels prevent T cells from being co-stimulated by competing with CD28 for binding to CD80 and CD86 on antigen-presenting cells [63]. These immune-inhibitory receptors also reduce glucose uptake by inhibiting PI3K/Akt/mTOR signaling (Fig. 2) [64, 65]. While much focus on metabolic reprogramming in stimulated T cells has been on the involvement of aerobic glycolysis, a new study has shown the significance of metabolic activities driven in mitochondria in T cell activation. In addition to energy production, the electron transport chain (ETC) also produces reactive oxygen species [66], which are vital for T cell activities [67, 68]. T cells lacking in Rieske iron-sulfur protein (RISP), a mitochondrial complex III subunit, display repressed in vitro and in vivo stimulation and expansion of antigen-specific T cells due to deficiencies in ROS signals derived from mitochondria [69]. While ROS is produced as a general by-product of mitochondrial metabolism, many studies have linked the metabolite succinate specifically to both ROS generation and HIF-1α activation in inflammation or injury settings [70, 71]. Since the activity of HIF-1α and ROS production by mitochondria is vital for reprogramming metabolic pathways after activation of naive T cells, there could be a speculation that succinate may aid in the events resulting to T cell activation from naïve cells. Among the TME's immune cells, tumor-associated macrophages (TAMs) and MDSCs are critical T cell activation regulators [72, 73]. MDSCs in the TME have been found to overexpress HIF-1α, which aids their differentiation as well [74]. HIF-1α induced by hypoxia was also found to be involved in the upregulation of PD-L1 on splenic MDSCs in the TME. The authors further demonstrated that MDSCs from the peripheral lymphoid organ (spleen) show differential expression of PD-L1 compared to MDSCs from the tumor site [54]. Overall, the studies reveal that selective inhibition of glycolytic intermediates, hypoxia inducing factors and checkpoint inhibitors could be a novel therapeutic approach for targeting MDSCs and thus improving immunotherapy in cancer patients.

### Non-invasive techniques for imaging T cell metabolism

#### PET Imaging of T cell metabolic pathways

For many decades, PET imaging has served as a robust non-invasive diagnostic tool for various diseases. More recently, PET has been investigated as an efficient tool to target activated T cells and T cell-related metabolic pathways in cancer. The most commonly used PET probes that target cellular metabolic pathways are 18F-fluorodeoxyglucose (^18^F-FDG) and 18F-fluorothymidine (^18^F-FLT). These probes are widely used to monitor treatment therapies' curative effects, including immunotherapies on tumors [75]. ^18^F-FDG provides information based on increased uptake of glucose in cells and helps depict metabolic abnormalities in various diseases [75]. In contrast, ^18^F-FLT serves as a marker of tumor cell proliferation that incorporates into the DNA of proliferating tumor cells and indicates the activity of thymidine kinase enzyme [75]. In a clinical study, high uptake of the ^18^F-FLT tracer was found in the lymph nodes (LNs) of melanoma patients who received DC therapy compared to the control LNs that received DCs without antigen [76]. Recently, there has been more focus on designing PET probes that use fluorinated deoxycytidine kinase (dCK) substrates for imaging T cells due to the extensive reliance of lymphoid and proliferating tissues on salvage pathways. Moreover, dCK is a rate-limiting enzyme in the deoxyribonucleoside salvage pathway, that is involved in the recycling of the DNA degradation products [77]. So, to measure the dCK activity in various immune disorders and cancers, a novel PET probe known as 2′-deoxy-2′-[18F]fluoro-β-D-arabinofuranosylcytosine, 1 ([^18^F] FAC) has been developed [78]. ^18^F-FAC was found to have greater specificity for lymphoid organs in a mouse model of anti-tumor immunity compared to other PET probes that are used to visualize nucleoside metabolism and glycolysis, which represent the major hallmarks in cancer [78]. One of the constraints in the clinical application of ^18^F-FAC is its rapid catabolism in humans due to the presence of the high concentrations of enzyme cytidine deaminase [79]. To overcome this issue, a group led by Antonios et al. developed a more clinically relevant dCK PET probe known as ^18^F-clofarabine (^18^F-CFA) [80]. The study showed that the combination of ^18^F-CFA PET and MRI proved efficient in distinguishing tumor progression and inflammation in patients with recurrent glioblastoma (GBM) treated with immune-based therapies (dendritic cell (DC) vaccine and/or PD-1 blockade) [80]. Another PET radiotracer that targets T cell related metabolic pathways is known as 2′-deoxy-2′-[18F]fluoro-9-β-D-arabinofuranosylguanine ([^18^F]F-AraG) [81]. Arabinosylguanine (AraG) is a deoxyguanosine analog that retains selective cytotoxicity for T-leukemic cells [82]. [^18^F]F-AraG is a substrate for cellular diacylglycerol kinase (dGK) and is found to accumulate in both activated and resting T cells [81]. In a study, [^18^F]F-AraG PET imaging enabled visualization of secondary lymphoid organs and allowed quantitation of increasing T cells in the cervical lymph nodes of a murine acute graft-versus-host-disease model [81]. Another PET tracer *trans*-1-amino-3-[^18^F] fluorocyclo-butanecarboxylic acid (anti-[^18^F]FACBC) is found to be commonly used to visualize prostate cancer (PCa) [83, 84]. In support of this, a study by Kanagawa et al. found higher accumulation of anti-[^18^F]FACBC in the lesional lumbar LNs than the non-lesional LNs in PCa LN metastasis (PLM) rats as compared to the acute lymphadenitis (AL) and chronic lymphadenitis (CL) rats [85].

#### PET Imaging of Immune checkpoints

Immune checkpoint inhibitors that target immune checkpoints such as CTLA4, PD-1 and PD-L1 have gained widespread attention in the field of immuno-oncology. CTLA4 acts as an “off” switch when bound to CD80 or CD86 membrane proteins and inhibits T cell activation. In many preclinical models, PET imaging probes have been developed to determine the expression of PD-1/PD-L1 and CTLA4 in the cancer tissues [86]. A study by Higashikawa et al. synthesized a CTLA4-targeting PET probe by using an anti-CTLA4 monoclonal antibody (mAb) conjugated with 64Cu-1,4,7,10-tetraazacyclododecane-N,N′,N″,N‴-tetraacetic acid (DOTA), and found a high accumulation of the probe in the CT26 tumor of the BALB/c mice [87]. Another preclinical study found an enhanced accumulation of the PET tracer, ^64^Cu-DOTA-ipilimumab in the CTLA4-expressing non-small cell lung cancer (NSCLC) tissues [88]. Another target for PET imaging is the T cell co-receptor CD3 which serves as a global T lymphocyte marker, and PET imaging of CD3 can provide a quantitative assessment of lymphocyte infiltration across tumors [89]. A study by Larimer et al. developed a CD3 PET imaging agent ^89^Zr-DFO-CD3 and found differential CD3^+^ T cell infiltration in CT26 tumor-bearing mice treated with anti-CTLA4 [90]. This study shows that CD3 PET imaging can help predict tumor responses to CTLA4 blockade.

On the other hand, PD-1/PD-L1 blockade serves as a promising therapeutic target in immunotherapy as the binding of PD-L1 to PD-1 receptor expressed on T cells causes suppression of T cell responses [91]. Initially, a PD-1 targeting ^64^Cu PET probe was developed using a murine mAb to detect PD-1 expressing murine TILs [92]. Furthermore, PET probes using nivolumab and pembrolizumab have been also developed to map the localization of TILs in murine models. A study showed the efficacy of ^89^Zr-Df-nivolumab probe for imaging PD-1 expressing T cells in a humanized murine model of lung cancer [93]. Another study demonstrated the use of ^89^Zr-labeled pembrolizumab PET probe in imaging PD-1 expressing TILs in a humanized melanoma murine model [94].

Moreover, other immunoPET probes such as ^64^Cu-NOTA-PD-1 and ^64^Cu-NOTA-PD-L1 efficiently enabled the visualization of PD-1 expressing TILs combined with immunoradiotherapy and PD-L1 expression in murine models of melanoma [95]. The study also showed that the expression of PD-L1 was more pronounced in the lungs after treatment with IFN-γ [95]. Moreover, a recent study showed the first-in-human assessment of the PET tracer ^89^Zr-atezolizumab (anti-PD-L1) in patients with NSCLC, triple-negative breast cancer (TNBC) and metastatic bladder cancer. The study found high but heterogeneous uptake of the tracer across different tumor types [96].

Thus, the above studies show the clinical potential and feasibility of PET imaging probes for studying T cell dynamics in cancer and can serve as facilitators of in vivo cancer immunotherapy.

#### Magnetic resonance imaging (MRI) and Magnetic resonance spectroscopy (MRS)

Non-invasive imaging techniques such as nuclear magnetic resonance (NMR) or magnetic resonance spectroscopy (MRS) have been shown to be useful in translating metabolic findings from preclinical models to humans [63, 97, 98]. MRS detects signals from nuclei with a specific magnetic property and helps identify a wide range of nuclei-containing metabolites such as ^1^H, ^13^C and ^31^P. The most widely used method for MRS is ^1^H MRS, which allows the detection of different amino acids, lipids, glycolytic intermediates, membrane-phospholipids and energy-related metabolites. MR spectroscopy is useful for the metabolic profiling of tumor as it can obtain comprehensive profiles of tumor and normal tissues [99, 100]. When used in patients or animal models, in vivo MRS is paired with magnetic resonance imaging (MRI) for the spatial analysis of metabolite signals [101]. The development of CEST MRI can help in imaging metabolites such as glutamate [102, 103], creatine [104], glucose [105], and lactate [106]. Such metabolites can be detected in T_EFF_ cells as T cells in the activated state undergo rapid glycolysis to help their energy needs. These metabolites’ levels can be monitored pre- and post-immune therapy to assess changes in the T_EFF_ cell density [107].

As discussed previously, the accumulation of lactate in tumors due to high dependence of cancer cells on glycolysis serves as an important biomarker in immunotherapy as higher lactic acid concentrations are found to impair T cell metabolism and function [52]. Also, tumor-derived lactic acid is found to impair and inhibit the differentiation and activation of T cells, monocytes and NK cells [108]. The increased production of lactic acid by lactate dehydrogenase A (LDHA) is found to impair the production of IFN-γ in tumor-infiltrating T cells and inhibits immunosurveillance, thereby contributing to tumor growth [52, 109, 110]. Lactate is also found to induce an immunosuppressive TME by modulating CD4^+^ T cell polarization, which sustains the progression of prostate carcinoma [111]. The conventional methods for detecting lactate in vivo are ^1^H MRS, ^13^C-labeled pyruvate infusion, and dynamic nuclear polarization (DNP) [106]. A recent study described for the first time an MRI method based on chemical exchange saturation transfer (CEST) to image lactate (LATEST) in mouse models of lymphoma [106]. The LATEST method was found to have two orders of magnitude higher sensitivity than the traditionally used ^1^H MRS method [106]. In an *in-vitro* study on stimulated T cells against CD3/CD28 observed ~ 12 time increase in lactate level and ~ 3 times increase in the alanine level compared to the non-stimulated T cells using ^1^H NMR [107]. Since lactate can be mapped in vivo using the LATEST imaging method, it has the potential to detect the T cells activity/ immunotherapy response in vivo in real-time. Therefore, lactate is not just a metabolite, but it is also an essential regulator of different molecular mechanisms underlying the development of an immunosuppressive TME. Thus, inhibition or suppression of lactate production by pyruvate dehydrogenase inhibitor such as dichloroacetate (DCA) [112] and buffering the pH in the TME [113] are found to restore T cell function and enhance the efficacy of immunotherapy-based cancer treatments [114].

Glutamate, a crucial metabolite of the glutaminolysis pathway, is implicated in cancer metabolism. A recently developed imaging technique known as glutamate-weighted chemical-exchange-saturation-transfer (GluCEST) allows high-resolution detection of glutamate and therefore, can be used as an efficient imaging technique to study cancer metabolism non-invasively. In support of this, a study used GluCEST for imaging human TNBC xenografts in mice treated with glutaminase (GLS) inhibitor, CB-839 and found reduced GluCEST signal in treated TNBC xenografts as compared to the vehicle control group [115]. Another study applied GluCEST at 7T (7T) to glioma patients and showed increased GluCEST contrast associated with diffuse aggressive gliomas [116].

Glutamine is a major substrate involved in cancer proliferation and investigating its metabolic flux is essential to understand its role in metabolic rewiring that controls the survival of neoplastic cells. Activated T cells increase glutamine uptake through the glutamine transporter (ASCT2/SLC1A5) and utilize extracellular glutamine for proliferation [59, 117]. Moreover, a recent study found that the loss of the GLS enzyme was found to reduce initial T cell activation and impaired the differentiation of Th17 cells [118]. Interestingly, the study also found that loss of GLS promoted differentiation and effector function of CD4 and CD8 T cells [118]. An interesting recent study by Thapaliya et al*.* demonstrated the role of glutamine metabolism in dysfunctional CD8^+^ T cells. The study found that glutamine is the major metabolic source for dysfunctional CD8^+^PD-1^+^TIM-3^+^ T cells in immune checkpoint inhibitors resistant melanoma [119].Glucose is another major contributor to cancer metabolism, as it is a nutrient that is required for the proliferation of both T cells and tumor cells and the increased consumption of glucose by tumors leads to a decrease in the amount of glucose levels within the TME and immune cells, creating competition for glucose between T cells and tumor cells for survival and proliferation. Measuring the conversion of glucose to lactate using LATEST can therefore be used to gain quantitative information about the metabolism of the cancer cells. Acetate is another major contributor to cancer cells' metabolism, as it is an essential source of acetyl CoA under conditions of hypoxia, and tumor growth is impaired by inhibition of acetate metabolism [120].

Interestingly, acetate is found to rescue T cell effector function by promoting histone acetylation, chromatin accessibility and enhancing IFN-γ production in glucose-restricted CD8^+^ T cells [121]. Thus, showing the efficacy of acetate as an alternative substrate to glucose in promoting T cell function under glucose-restricted conditions. Acetyl-CoA is involved in the synthesis of fatty acids known as lipogenesis, which proves necessary for cell growth and survival under nutrient-poor conditions. Studies have reported increased amounts of acetyl CoA labeled with the ^13^C-acetate tracer in tumor cells under hypoxic and nutrient-poor circumstances, indicating that acetate metabolism is related to metabolic stress conditions [122]. Acetate is a specific biomarker in glial cells metabolism, and several ^13^C-labeled acetate studies have shown the evidence of this metabolite in normal brain metabolism [123, 124]. Brain tumors are found to be capable of oxidizing acetate, as demonstrated by a study in which orthotopic brain tumors oxidized [1,2-^13^C] acetate in the TCA cycle, indicating that this adaptation could be due to the high energy demands needed by tumors for growth [125].

**Fig. 3:**
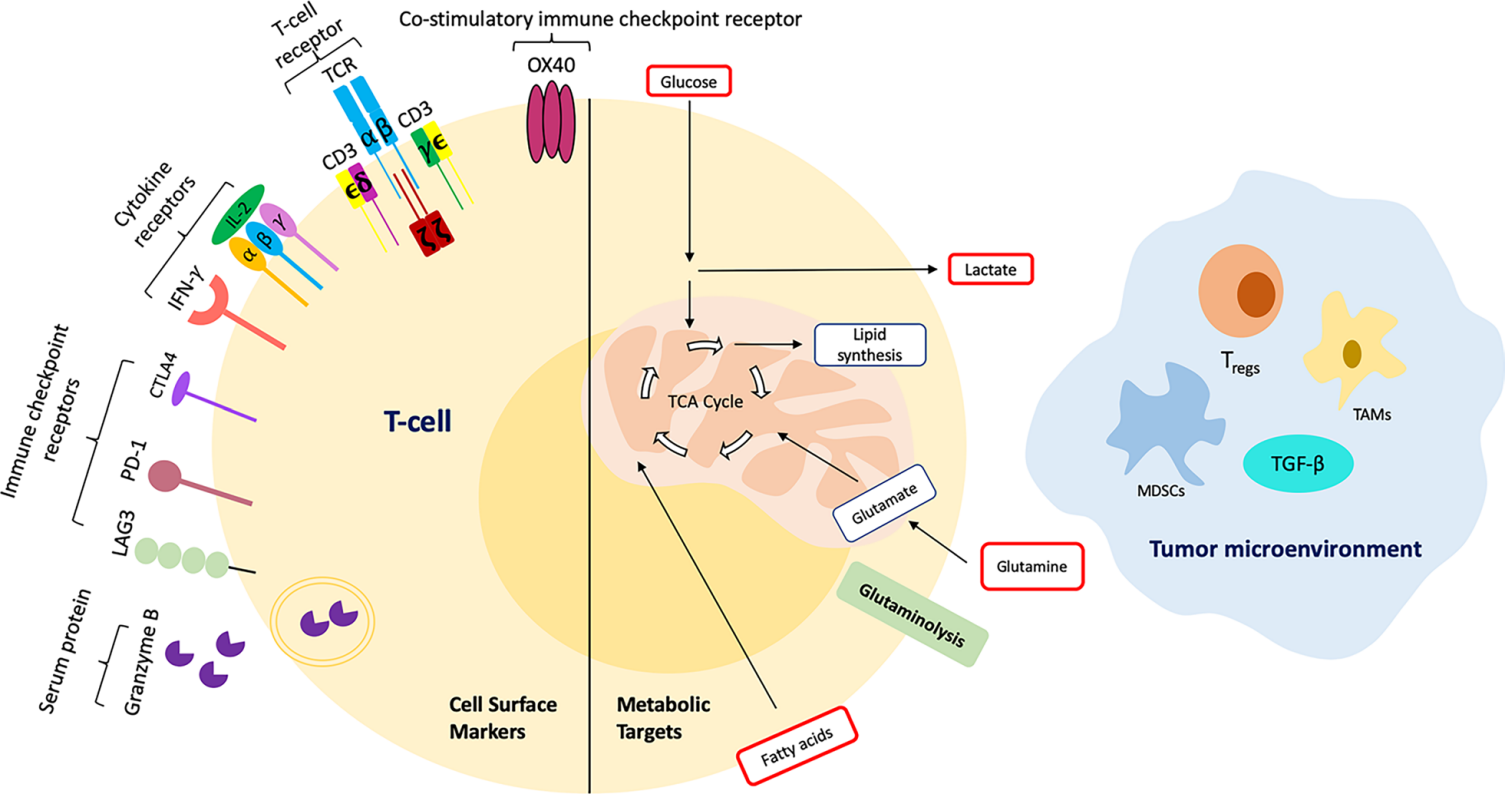
Imaging targets in T cells and tumor microenvironment (TME). Different Tcell surface markers such as T cell receptors, cytokine receptors, immune checkpoint receptors and serum proteins and various metabolites such as glutamine, glucose, lactate, and fatty acids can serve as targets for imaging T cell metabolism and effector functions. Also, imaging other components in the TME, such as MDSCs, TAMs, T_regs,_ and cytokines such as TGF-β, can enhance the efficacy of monitoring immunotherapies

Imaging specific T cell surface markers, metabolic targets, and other TME components can help understand the significance of T cell metabolism in cancer immunotherapy and track metabolic pathways disrupted in cancer cells’ metabolism that can help improve immunotherapy for cancer (Fig. 3).

## Conclusion

While immunotherapy holds great promise, the selection of the right treatment for a particular patient is crucial. There is an unmet need for non-invasive biomarkers that can reliably evaluate T cells activity in TME and guide treatment decisions in an overall effort to restore active antitumor immune responses. The challenge in identifying suitable biomarkers for the selection of drug-responsive patients is the dynamic change in targets for immunotherapy that occurs during the interaction between tumor cells and immune cells within TME. A clear understanding of tumors' metabolic environment is a key factor in determining whether there are successful anti- tumor immune responses, whether lymphocytes and drugs can reach the tumor, and how best to suit a tumor with specific immunotherapy. Molecular imaging methods may allow for the non-invasive detection of multicellular components of tumor microenvironments to better predict immunotherapy response and monitor changes in tumor composition during treatment. Non-invasive approaches that target the T cell metabolism can provide imaging biomarkers that may be used for cancer immunotherapy response assessment and monitoring. Imaging the various metabolites involved in cancer and tracking these metabolites pre- and post-immunotherapy will help to unravel the complex metabolic profile of tumors.

Furthermore, imaging the function of T cells that are adoptively transferred will help to understand the mechanisms by which T cells interact with tumor cells. Such methods require more clinical development and validation. Also, exploring and developing non-invasive biomarkers with in vivo imaging to monitor T cell responses during immunotherapy is an essential requirement for the success of anti-cancer immunotherapy. As researchers identify additional molecules that regulate immune checkpoints, the development of approaches to multiparametric imaging of immune environments will likely help establish better predictive biomarkers.

## Data Availability

Not applicable.
